# The accuracy of magnifying narrow band imaging (ME-NBI) in distinguishing between cancerous and noncancerous gastric lesions: A meta-analysis

**DOI:** 10.1097/MD.0000000000009780

**Published:** 2018-03-02

**Authors:** Fan Zhou, Liucheng Wu, Mingwei Huang, Qinwen Jin, Yuzhou Qin, Jiansi Chen

**Affiliations:** Department of Gastrointestinal Surgery, Affiliated Tumor Hospital of Guangxi Medical University, Guangxi, China.

**Keywords:** gastric lesions, meta-analysis, narrow-band imaging

## Abstract

**Background:**

Previous clinical trials have demonstrated the diagnostic accuracy of magnifying narrow-band (ME-NBI) for gastric cancerous lesions, but the results are inconsistent. The purpose of this meta-analysis is to investigate the accuracy of ME-NBI in distinguishing between cancerous and noncancerous gastric lesions.

**Methods:**

Systematic literature searches were conducted until October 2016 in PubMed, Embase by 2 independent reviewers. Meta-analysis was performed to calculate the pooled sensitivity, specificity. Two authors independently evaluated studies for inclusion, rated methodological quality, and abstracted relevant data. Meta-analytic method was used to construct summary receiver operating characteristic curves, and pooled sensitivity, specificity were calculated.

**Results:**

Nine studies enrolling 5398 lesions were included. The pooled sensitivity, specificity were 88% (95% confidence interval [CI]: 78–93%), 96% (95% CI: 91–98%), respectively. The area under the curve (AUC) was 0.97. There was a large heterogeneity between the included studies. Studies with lesions ≤ 10 mm still had a high pooled sensitivity of 81% (95% CI: 73–90%) and specificity of 97% (95% CI: 95–100%). Studies which analyzed resected specimens had a sensitivity of 91% (95 CI: 82–99%) and specificity of 88% (95% CI: 83–94%), and studies which analyzed biopsied specimens had a sensitivity of 85% (95 CI: 74–96%) and specificity of 99% (95% CI: 98–99%).

**Conclusions:**

ME-NBI is highly accurate and consistent to distinguish between gastric cancerous and noncancerous lesions.

## 1 Introduction

Gastric cancer (GC) has a high incidence and mortality all over the world.^[1]^ Although the death rate is gradually declining, the rate of early diagnosis is still low. If gastric cancer can be early spotted, we will be able to prolong the survival time.^[2]^

Conventional white-light imaging (C-WLI) has been used for many years, but the accuracy in diagnosing gastric cancer is still low.^[3]^ Many studies have indicated that the sensitivity of C-WLI varied from 40% to 60% and specificity varied from 67.9% to 94.3%.^[3]^ And differentiation between cancerous and noncancerous lesion is especially difficult using C-WLI alone. However, the appearance of magnifying endoscopy with narrow-band imaging (ME-NBI) has been changing this scenario.^[4]^ In ME-NBI, blue (415 nm) and green (540 nm) light is selectively emitted to tissues through a narrow-band filter at the tip of the scope. Both the blue and the green light are absorbed by hemoglobin, while the green light reflects at the shallower level and preferentially visualizes the superficial capillary network, whereas the blue light penetrates deeper and enables visualization of the vasculature at the subsurface level. Then it can clearly display the visualization of the superficial mucosal structures and vascular structures. The VS (vascular and surface pattern) classification proposed by Yao et al is the most commonly used classification to characterize superficial gastric lesions.^[3,5]^ Although it has been established that ME-NBI was more accurate than C-WIL endoscopy in identifying early gastric cancer, but its sensitivity and specificity differed from study to study. The reasons are as follows: the characteristics of gastric lesions are different, the size of gastric lesions are different. Although 2 previous meta-analyses were carried out to address this problem, but both studies mainly tried to compare the diagnostic efficacy of C-WLI with ME-NBI and included studies that used different diagnostic criteria such as ABC, VS, and Type A-E.^[6,7]^ So we performed this meta-analysis to systematically investigate the diagnostic performance of ME-NBI in differentiating between gastric cancerous and noncancerous lesions.

## 2 Materials and methods

This meta-analysis was reported according to the preferred reporting items for systematic reviews and meta-analyses guidelines. All analyses were based on previous published studies, thus no ethical approval and patient consent are required.

### 2.1 Search strategy

We systematically searched relevant literature until June 2017 in PubMed, Medline, and Embase. The search terms were as following: (“narrow band” OR “narrow band imaging” OR “NBI”) AND (“gastric cancer” OR “gastric carcinoma” OR “gastric neoplasm” OR “stomach cancer” OR “stomach carcinoma” OR “stomach neoplasm”). Computerized literature search was augmented by manually reviewing the reference lists of identified studies, abstracts from recent conference proceeding. We included studies published in any language.

### 2.2 Selection criteria

The relevant literature must meet the following criteria: ME-NBI was used for the diagnosis of gastric lesion; true-positive (TP), false-positive (FP), true-negative (TN), and false-negative (FN) were reported or could be calculated; vessel plus surface (VS) classification system was used; the diagnostic gold standard was the pathology. Articles that conform to the following will be excluded: the sample data were incomplete, and the number of TP, FP, TN, FN could not be obtained; diagnostic criteria used in the study was not VS classification system; review articles, case reports, editorials, comments.

### 2.3 Qualitative assessment

The studies included in this paper are evaluated by the Quality Assessment of Diagnostic Studies-2 (QUADAS-2).^[8]^ It is used for systematic reviews of diagnostic accuracy studies. QUADAS-2 tool consists of 4 parts: patient selection, index test, reference standard, flow and timing. All parts are required during the assessment. The relevant questions of each part will be answered with “yes,” “no,” or “unclear,” and corresponding to the bias risk rating can be judged as “low,” “high,” or “unclear.” If all the answers to a range of symbolic questions are “yes,” they can be assessed as low bias risk. In contrast, if all the answers are “no,” then bias exists. The entire evaluation process was performed independently by 2 reviewers (FZ and LW).

### 2.4 Statistical analysis

The pooled sensitivity, specificity, and likelihood ratio are calculated by random effects model, and illustrated by forest map. To quantitatively summarize study results, we used a meta-analytic method to construct summary receiver operating characteristic curves (SROC).^[9]^ SROC illustrate the trade-off between sensitivity and specificity. It is assumed that each individual study represents a unique point on a common ROC curve. The maximum joint sensitivity and specificity is the point on a symmetrical ROC curve that is intersected by a diagonal line that runs from the bottom right corner to the top left corner of the ROC diagram. This point is a global measure of test accuracy, similar to the area under the ROC curve (AUC). So we used AUC value which is between 1 and 0.5 in this meta-analysis. If the AUC is closer to 1, it indicates better diagnostic performance. While an AUC with 0.5 to 0.7 indicates low accuracy, an AUC with 0.7 to 0.9 indicates a certain accuracy, an AUC more than 0.9 indicates good accuracy. In diagnostic studies, heterogeneity in sensitivity and specificity can result from many causes related to definitions of the test and reference standards, operating characteristics of the test, methods of data collection, and patient characteristics. Covariates may be introduced into a regression with any test performance measure as the dependent variable. While at the same time, subgroup analysis according to these covariates can be carried out. We can also get the *I*^2^ value to express the heterogeneity. A *I*^2^ greater than 50% was found to have heterogeneity. Formal testing for publication bias will be conducted by a regression of diagnostic log odds ration against 1/sqrt (effective sample size), weighting by effective sample size, with *P* < .10 for the slope coefficient indicating significant asymmetry.^[10]^ All data analyses were conducted by STATA version 11.0.

## 3 Results

### 3.1 Search results

**Figure 1:**
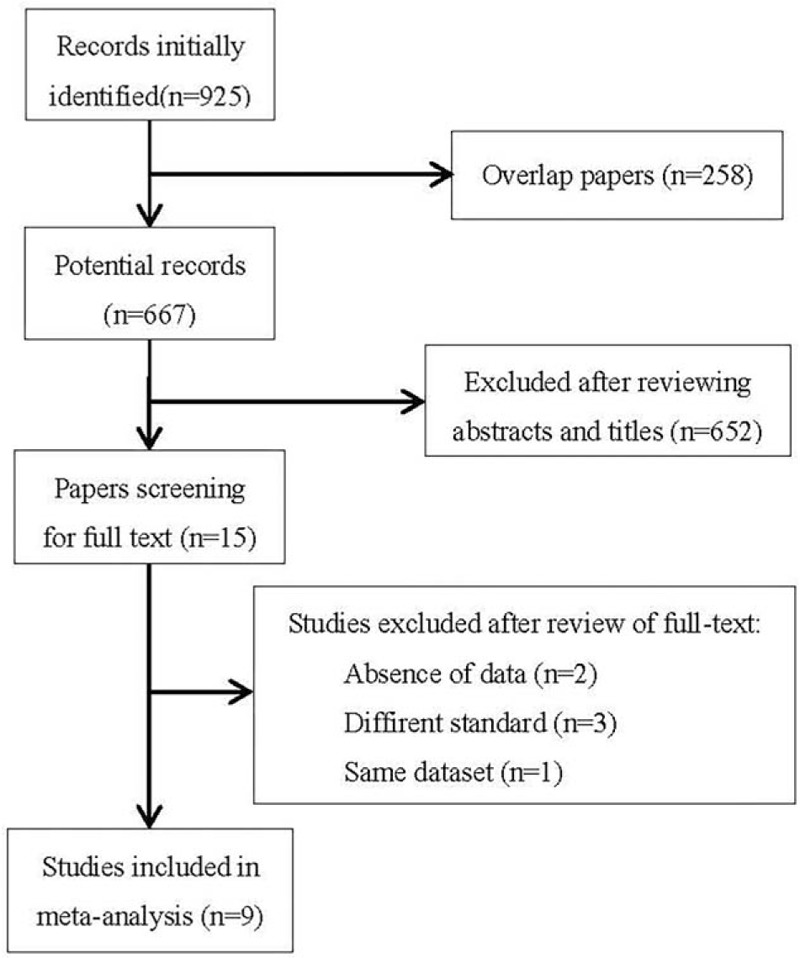
Flow chart shows the selection process.

After searching PubMed, Medline, and Embase, 925 potentially relevant studies were initially identified. We excluded 258 studies because of duplication, then another 652 studies after scanning their titles and abstracts. Thus, 15 potentially eligible studies were subsequently appraised. After retrieval of full text, we excluded studies which did not use VS classification system,^[11–13]^ did not present sufficient data to permit calculation of sensitivity and specificity,^[14,15]^ or presented data that was reported elsewhere.^[16]^ At last, 9 studies met the inclusion criteria and were included.^[3,17–24]^ The study flow diagram is shown in Fig. 1. The characteristics of these 9 studies are summarized in Table 1.

**Table 1:**
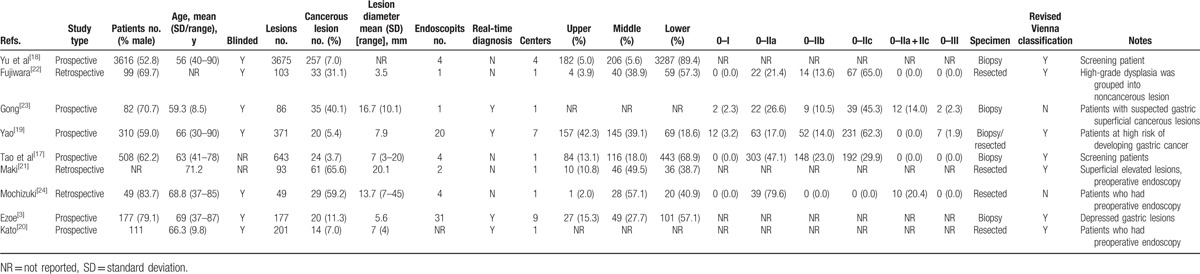
Basic characteristics of the included studies.

### 3.2 Quality assessment

Of those 9 included studies, 6 were prospective.^[3,17–20,23]^ The number of lesions ranges from 49 to 3675, all of these studies were carried out in Japan and China. All the studies adequately described the technical aspects of performing ME-NBI. While Yu et al^[18]^ and Tao et al^[17]^ enrolled screening patients in their studies, Fujiwara et al^[22]^ only enrolled patients with minute gastric lesions (≤5 mm), Gong et al^[23]^ enrolled patients with suspected superficial cancerous lesions, Yao et al^[19]^ only included patients with superficial depressed lesions ≤10 mm who are at risk of developing gastric cancer, Maki et al^[21]^ just enrolled patients with superficial elevated lesions, Mochizuki et al^[24]^ only enrolled patients diagnosed as having adenomas by forceps biopsy, Ezoe et al^[3]^ enrolled patients with depressed gastric lesions ≤ 10 mm that were newly detected and undiagnosed, and Kato et al^[20]^ enrolled patients with lesions that were recognized or suspected of being cancerous. Thus, the characteristics of the patients varied among different studies. The mean size of lesion diameter ranges from 5.6 to 20.1 mm. Although all these studies employed VS classification system, but Yao et al made endoscopic diagnosis according to degree of certainty and need for biopsy,^[19]^ which is totally different from the rest of these studies. And this specific study reported a very low sensitivity value of 60%.^[19]^ A real-time diagnosis of ME-NBI was made in 4 studies,^[3,19,20,23]^ for the other 5 studies the diagnosis was made later while reviewing recorded endoscopic images.^[17,18,21,22,24]^ While pathological diagnosis was used as the criterion standard, but 4 studies used biopsy specimens,^[3,17,18,23]^ 4 studies used resected specimens,^[20–22,24]^ and 1 used both biopsy and resected specimens.^[19]^ Lesions diagnosed as high-grade neoplasia (category 4) were designated as noncancerous in 3 studies,^[22–24]^ while they were designated as cancerous in the other 6 studies.^[3,17–21]^ All included studies were evaluated by QUADAS-2 (Table 2).

**Table 2:**
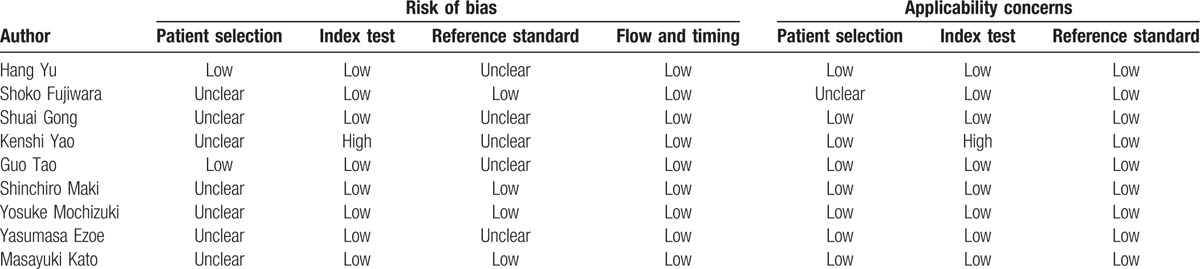
Quality of studies using the quality assessment of diagnostic accuracy studies.

### 3.3 Analysis results

**Figure 2:**
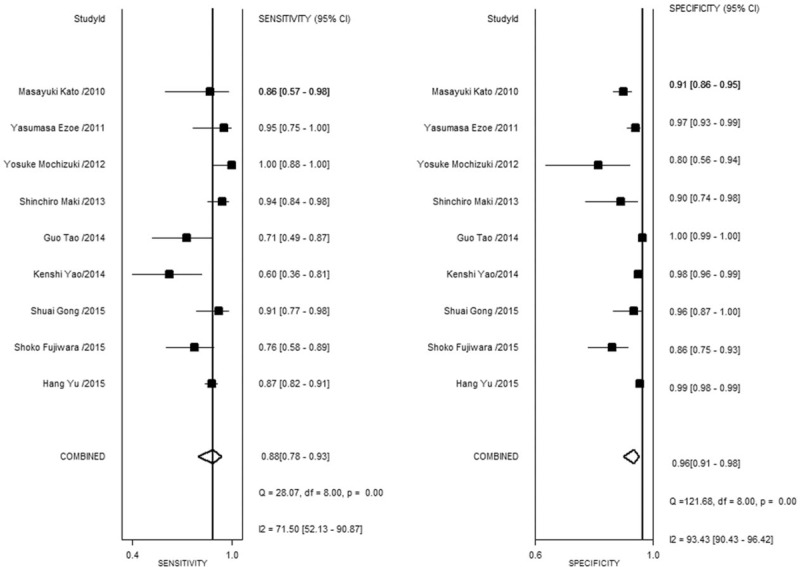
Forest plot showing pooled sensitivity and specificity of ME-NBI for gastric lesions.

**Figure 3:**
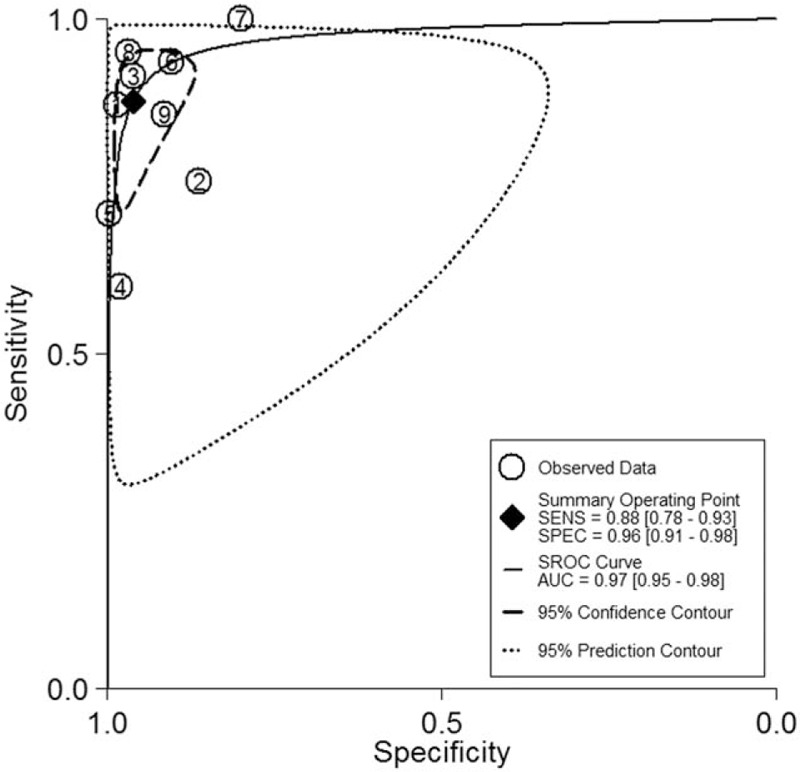
Summary receiver operating characteristic curve showing the diagnostic accuracy of ME-NBI for gastric lesions.

Nine studies with a total of 5398 lesions were included in this meta-analysis. The sensitivity of ME-NBI for distinguishing between cancerous and noncancerous lesions ranged from 60% to 95%, while specificity ranged from 80% to 99%. The pooled sensitivity and specificity were 88% (CI, 78–93%) and 96% (CI, 91–98%), respectively (Fig. 2); Fig. 3 shows the SROC curve and 95% CIs, and the value of AUC was 0.97. Since Yao et al made endoscopic diagnosis according to degree of certainty and need for biopsy which is different with the rest of the studies included. When this study was excluded from meta-analysis, the pooled sensitivity and specificity were 89% (CI, 82–94%) and 96% (CI, 90–98%), respectively.

**Figure 4:**
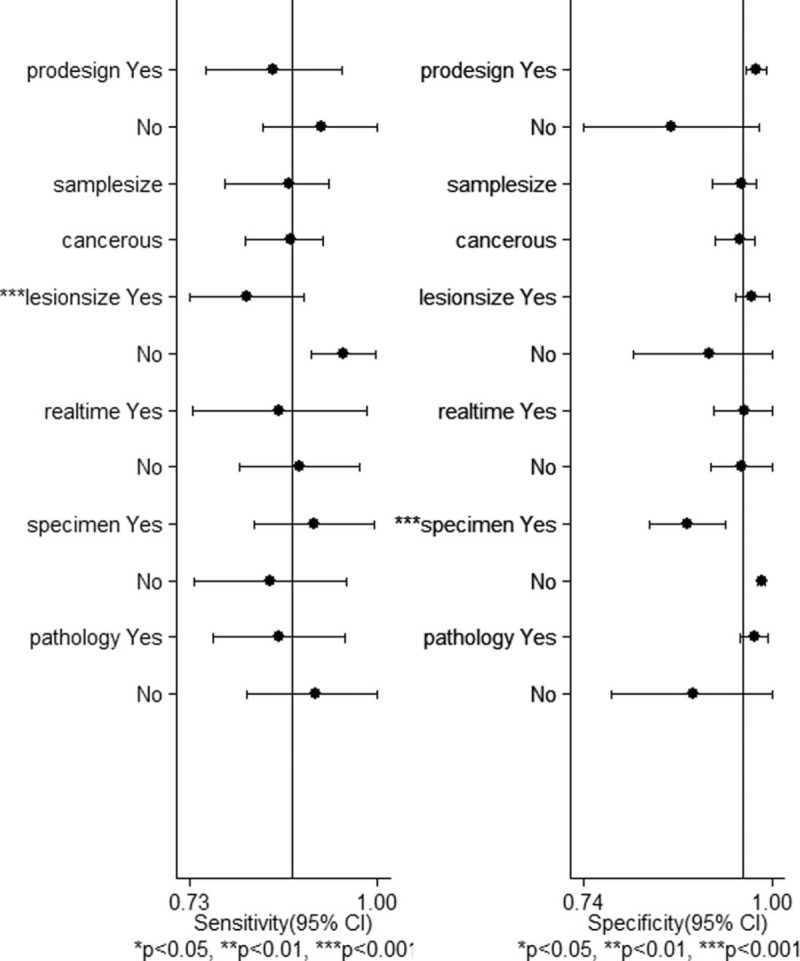
Meta-regression and forest plot showing pooled sensitivity and specificity of subgroup analysis on diagnostic accuracy of ME-NBI for gastric lesions. Prodesign Yes: studies were prospective. Samplesize: number of lesions. Cancerous: the percentage of cancerous lesions. Realtime Yes: real-time diagnosis. Specimen Yes: resected specimen. Pathology Yes: high-grade neoplasia was designated as noncancerous.

Meta-regression showed diagnostic accuracy was better in studies which were prospective compared with the remaining studies which were retrospective (*P* = .03), it was better in studies with higher proportion of cancerous lesions compared with studies with lower proportion of cancerous lesions (*P* = .02), it was better in studies which the mean size of the lesions > 10 mm compared with studies which the mean size of the lesions ≤ 10 mm (*P* = .04), it was better in studies which the specimens were resected compared with studies which the specimens were biopsied (*P* = .00). The pooled sensitivity and specificity for 6 studies that were prospective were 85% (CI, 75–95%) and 98% (CI, 96–99%), respectively. The pooled sensitivity and specificity for 3 studies with the mean size of the lesions > 10 mm were 95% (CI, 90–100%) and 91% (CI, 91–100%), respectively; while they were 81% (CI, 73–90%) and 97% (95–100%) respectively for 6 studies with the mean size of the lesions ≤ 10 mm. The pooled sensitivity and specificity for 5 studies which the specimens were biopsied were 85% (CI, 74–96%) and 99% (CI, 98–99%), respectively (Table 3, Fig. 4).

**Table 3:**
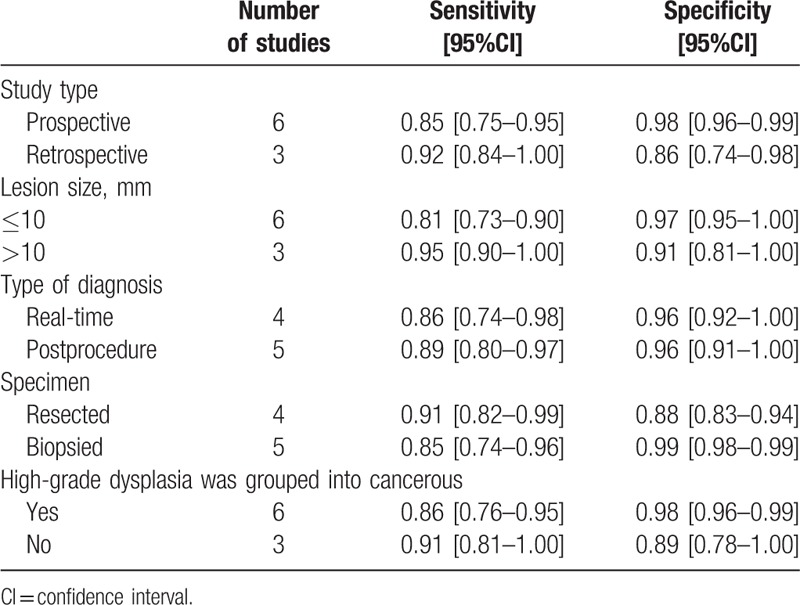
Subgroup analysis on diagnostic accuracy of ME-NBI in distinguishing between cancerous and noncancerous gastric lesions.

**Figure 5:**
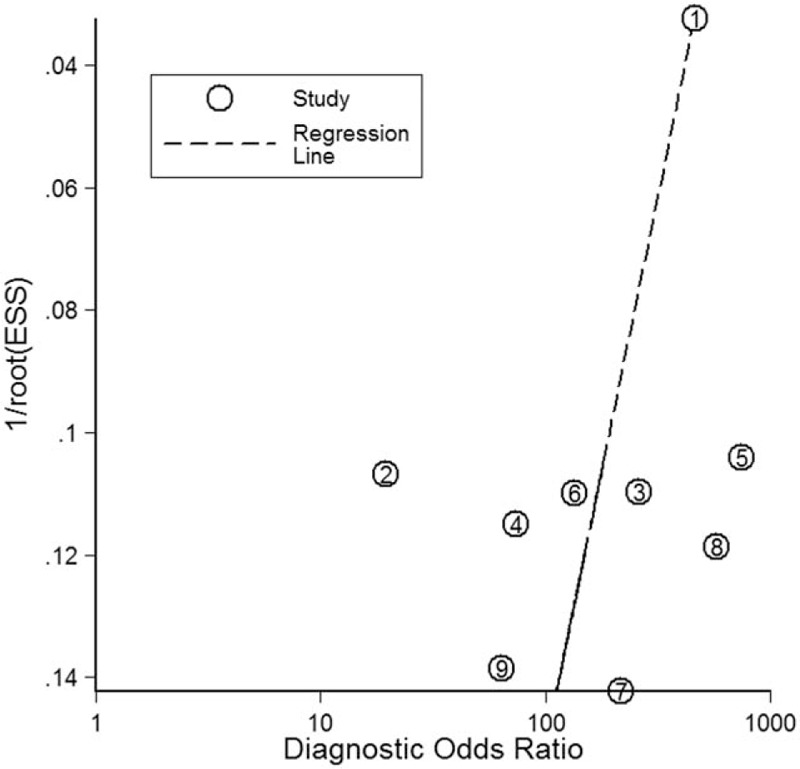
Deeks’ funnel plot for publication bias.

To verify publication bias, we also produced Deek's funnel plots (Fig. 5), while funnel plot did not suggest evidence of publication bias (*P* = .10).

## 4 Discussion

This meta-analysis demonstrated that ME-NBI offers a high sensitivity of 88% and specificity of 96% in distinguishing between cancerous and noncancerous gastric lesions, which were higher than those of C-WLI. Thus, ME-NBI is definitely better compared with C-WLI in distinguishing between cancerous and noncancerous lesions, which has been the standard endoscopic examination for the identification of suspicious lesions.^[3]^

By means of ME-NBI examination, one can visualize the microsurface and microvascular architecture immediately, which is not like chromoendoscopy that requires the administration of intravital dyes such as acetic acid or indigo carmine.^[25]^ So, ME-NBI is much more time-efficient and convenient. Since the VS classification is the most commonly used structured classification to characterize superficial gastric lesions, so we only included studies using this classification in this meta-analysis. Although pathology was the reference standard, but the actual grouping was different across studies as we have mentioned before. Meta-regression did not suggest that this would affect the diagnostic accuracy (*P* = .16), it is best all the future study use the revised Vienna classification and group high-grade dysplasia into cancer.^[26]^ But this meta-analysis revealed that the characteristics of the gastric lesions such as the type and the size of the lesions differed from each other, which may contribute to heterogeneity between studies. We also demonstrated that the diagnostic performance of ME-NBI was influenced by the size of gastric lesions and the specimens, while the later finding was totally unexpected. Our results showed that the diagnostic sensitivity was 81% in gastric lesions with a diameter ≤ 10 mm, the specificity for these lesions was 97%. Given the incidence of minute gastric cancer is low (32 of 30,725 upper gastrointestinal endoscopy cases),^[22]^ when a lesion is less than 10 mm and considered noncancerous by experienced endoscopist using ME-NBI, a negative biopsy could be probably avoided. When we only included studies which analyzed resected specimens, the specificity decreased from 96% to 88%. It is possible that biopsy alone may lead to some misdiagnoses which happen to be cancerous. Future studies investigating ME-NBI should really have the gastric lesion resected rather than just have them biopsied. Whether it is real-time diagnosis or postprocedure diagnosis, it does not influence the diagnostic accuracy of ME-NBI. It implies that the VS classification can be easily employed and it is consistent, and its clinical applicability is feasible. At the same time, studies have also suggested that ME-NBI has great reproducibility for the endoscopic diagnosis of gastric cancers. The κ value for interobserver variability and intraobserver variability for ME-NBI examination of gastric lesions were 0.56 and 0.65, respectively, both suggested moderate agreement.^[22]^

Making a differential diagnosis of small gastric lesions between noncancerous and cancerous used to be one of the limitations of C-WLI. Endocytoscopy and confocal laser microendoscopy have been developed to achieve “endoscopic pathology,”^[27–29]^ but unlike ME-NBI, these require intravenous or endoluminal administration of a day or fluorescent reagent, which limits their clinical use. On the contrary, ME-NBI enhances the quality of microstructure imaging without the need for dye or fluorescent staining. And its diagnostic accuracy could be significantly improved when the combination of C-WLI and ME-NBI was used.^[30]^ Since the pathological findings such as histological type, are needed for a diagnosis of cancer. Then a biopsy cannot be omitted in clinical practice. However, sometimes we are unable to take biopsies from a suspicious lesion because the patient is on intensive antithrombotic therapy which cannot be discontinued. In such cases, when the endoscopic diagnosis by ME-NBI is noncancerous, a negative biopsy could be avoided. In the case of the diagnosis is cancerous, we should perform resection after heparinization. Thus, ME-NBI could minimize the number of biopsies of noncancerous lesions taken. In fact, ME-NBI is cost-effective because it could reduce the number of biopsies required to detect a cancer in screening endoscopy.^[19]^ Besides, ME-NBI can distinguish the cancerous mucosa from surrounding tissues. Therefore, endoscopists can delineate the exam margin of gastric lesions so that successful endoscopic or surgical resection of the cancerous lesions is performed.

There are some limitations of this meta-analysis. First, the heterogeneity between studies was obvious and large, such as the patients’ risk of gastric cancer, the lesion size, the morphological type, and so on. Although we tried to limit heterogeneity through subgroup analysis, it is impossible to eliminate all the existing heterogeneity while we were calculating the pooled sensitivity and specificity. Second, it was known that experiences did influence the diagnostic accuracy of ME-NBI for gastric cancer,^[31,32]^ but definition of “experienced endoscopist” was either undescribed or unclear. Therefore, future studies should define it in a standard or an objective way.

In conclusion, ME-NBI has a high diagnostic accuracy in distinguishing between cancerous and noncancerous gastric lesions. This technique could enable endoscopists to observe gastric lesions more clearly and identify suspected lesions more accurately. Negative diagnosis by ME-NBI could avoid unnecessary biopsies, especially in patients who are weak or at risk of bleeding. ME-NBI may be a promising modality for endoscopic pathology in a standard clinical setting.^[33]^ Future studies should focus on whether the usage of ME-NBI could improve survival in randomized control trials.
